# Muscarinic acetylcholine receptor 3 mediates vagus nerve-induced gastric cancer

**DOI:** 10.1038/s41389-018-0099-6

**Published:** 2018-11-21

**Authors:** Linjun Wang, Jianghao Xu, Yiwen Xia, Kai Yin, Zheng Li, Bowen Li, Weizhi Wang, Hao Xu, Li Yang, Zekuan Xu

**Affiliations:** 1 Department of Gastric Surgery, The First Affiliated Hospital of Nanjing Medical University, Nanjing, Jiangsu 210029 China; 2 Department of General Surgery, Affiliated Hospital of Jiangsu University, Zhenjiang, Jiangsu 212000 China; 3 Collaborative Innovation Center for Cancer Personalized Medicine, Nanjing Medical University, Nanjing, Jiangsu 210029 China

## Abstract

Increasing evidence shows that the vagus nerve plays an important role in tumourigenesis. However, the effects and underlying mechanisms of the vagus nerve on gastric cancer (GC) development have not been established. In this study, we performed a unilateral truncal vagotomy at the subdiaphragmatic level in a mouse xenograft GC model to study the effects of the vagus nerve on GC development. Gene microarray analysis was used to explore the mechanism underlying this process. Significantly altered genes and pathways were analysed by Kyoto Encyclopaedia of Genes and Genomes analysis tool. We also detected muscarinic acetylcholine receptor 3 (M3) mRNA and protein levels by quantitative real-time polymerase chain reaction and immunohistochemical staining in mouse stomach tissue. To further confirm the functional role of M3, an in vivo M3 selective antagonist (darifenacin) assay was applied. Finally, we determined the M3 protein levels in human GC tissues and paired non-cancerous gastric tissues by immunohistochemical staining. We found that the surgical vagotomy inhibited the development of GC in an orthotopic xenograft mouse model. Further analysis showed that multiple signalling pathways participated in this process and that M3 was a key factor in these pathways. We established that the M3 mRNA and protein levels decreased in the vagotomy group relative to the sham group. We also demonstrated that the M3 antagonist suppressed the development of GC. Finally, we revealed that M3 protein level was up-regulated in human GC tissues. In conclusions, we revealed the functional role of M3 on mediating the effects of the vagus nerve on GC. Our study contributes to understanding the mechanism underlying the interaction between GC and the vagus nerve and may help to identify new therapeutic targets for GC.

## Introduction

Nerves are traditionally regarded as transmission structures for electrical and chemical signals. However, increasing evidence has shown that they play an important role in tissue repair, regeneration and tumourigenesis^1^. The interaction between nerves and cancer has been established in several tumour types. Neural invasion of cancer cells contributes to tumour development by providing a route for cancer cell dissemination in pancreatic cancer^2,3^, prostate cancer^4^, colon cancer^5^, rectal cancer^6^ and gastric cancer (GC)^7,8^. Further analysis showed that autonomic nerve density is associated with tumour proliferation in prostate cancer. Sympathetic nervous fibres play important roles in the initial phases of cancer development by promoting tumour cell survival, while cholinergic fibres from the parasympathetic nervous system infiltrate the tumour tissues and play major roles in tumour cell invasion, migration, and distant metastases^9^.

GC is one of the most common cancers worldwide, with 5-year survival rate less than 25%^10^. A partial resection of stomach with denervation promotes the tumour-related progress in the remnant stomach^11^. In human and mouse models of GC, incidence of GC in the lesser curvature (approximately 80% of tumours) was higher than in the greater curvature^12–14^. The vagus nerve exerts a prominent role in the innervation of the stomach, and it is mainly distributed in the lesser curvature, suggesting that tumour prevalence correlates with the distribution of nerve fibres. Zhao et al. showed that the densities of vagus nerve fibres and terminals correlate with tumour prevalence in the murine stomach, and surgical or pharmacological denervation inhibited gastric tumourigenesis in three independent mouse models of GC^13^. However, the effects and underlying mechanisms of the vagus nerve on the development of GC have not been established.

Muscarinic acetylcholine receptor 3 (M3), a G-protein coupled receptor, is one acetylcholine receptor that regulates many fundamental activities of central and peripheral nervous system^15^. Recent researches have identified the function of M3 receptor in several cancers, including GC. Song reported that M3 promotes small cell lung cancer proliferation in vitro^16^. Raufman revealed that M3 genetic ablation suppresses the proliferation and neoplasia of murine colon epithelial cell^17^. We previously demonstrate that M3 regulates cell proliferation induced by acetylcholine and promotes apoptosis in GC^18^. Whether M3 regulates the effects of the vagus nerve on GC is still unknown.

In this study, we found that a unilateral truncal vagotomy inhibited the development of GC in a mouse xenograft GC model. Further analysis showed that multiple signalling pathways participate in this process, and M3 is a key factor in these pathways. M3 mediates the effects of the vagus nerve in promoting GC development.

## Results

### Establishment of a nude mouse model to explore the effects of the vagus nerve on GC

**Fig. 1:**
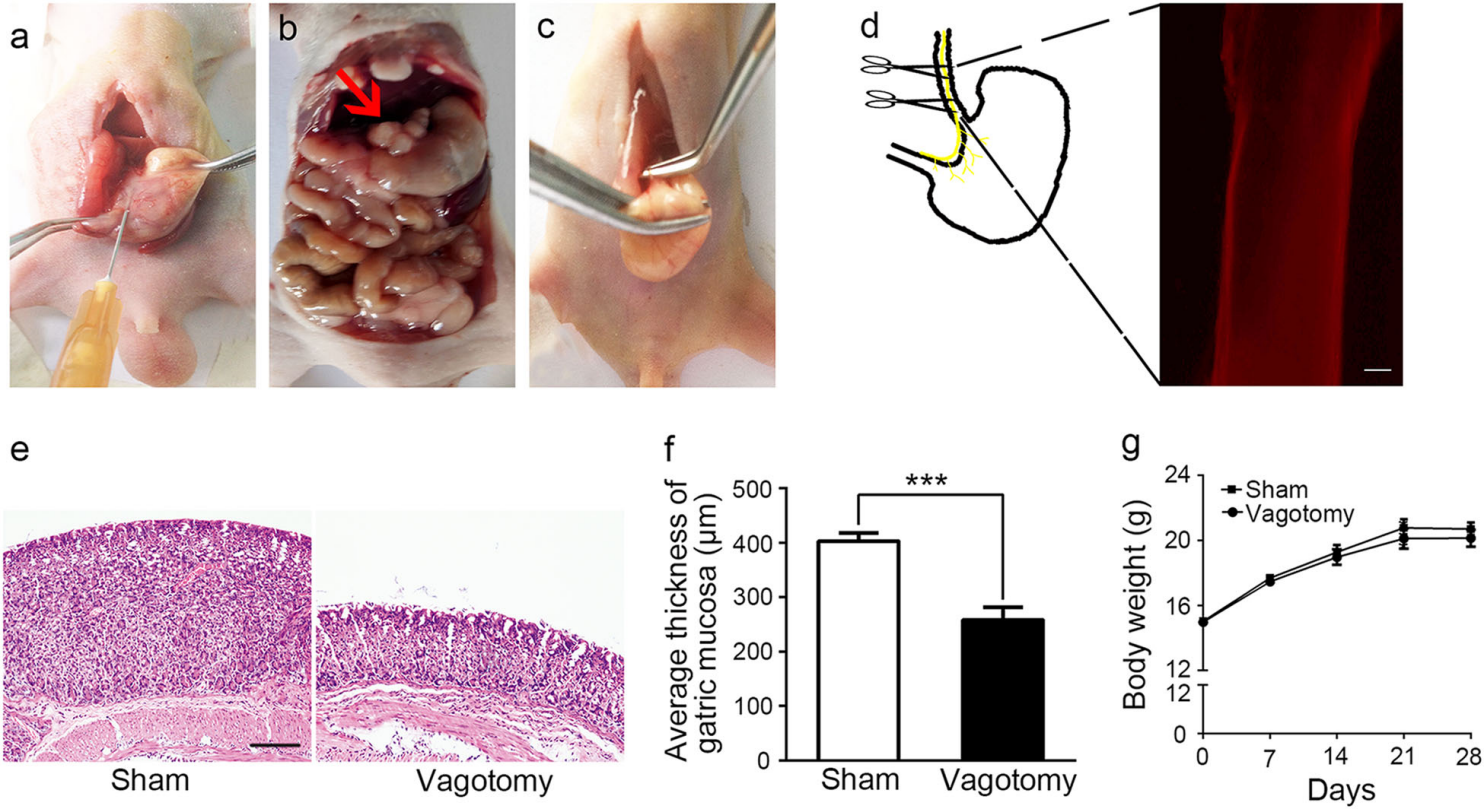
Establishment of vagotomy in an orthotopic xenograft nude mouse model. **a** Injection of luciferase-labelled MGC-803 cells into the subserosa of the lesser curvature of the mouse stomach. **b** Formation of a xenograft tumour in a mouse stomach (red arrow points to a xenograft tumour). **c** Transection of the left trunk of the vagal nerve at the subdiaphragmatic level. **d** Immunohistochemical (IHC) analysis of PGP9.5 in the dissected vagus nerve (original magnification, ×100; scale bar = 100 μm). **e** Haematoxylin and eosin staining (HE) of mouse gastric mucosa in the sham and vagotomy groups (original magnification, ×100; scale bar = 100 μm). **f** Average thickness of mouse gastric mucosa in the sham and vagotomy groups. **g** Body weight of the mice in the sham and vagotomy groups

To explore the effects of the vagus nerve on the development of GC, we used an orthotopic xenograft nude mouse model with luciferase-labelled MGC-803 cells (Fig. 1a, b), which generated in vivo bioluminescence imaging of the xenograft in situ. We then transected the left trunk of the vagus nerve at the subdiaphragmatic level (Fig. 1c), without impairing the overall functional capacity of the stomach, circulating gastrin levels or gastric acid output^19^. To confirm that the transected tissue was nerve fibre, we removed the nerve and immunostained it with a specific neuronal marker (PGP9.5). The resected nerve showed positive nerve staining (Fig. 1d, e). We also measured the body weight and thickness of the gastric mucosa of the mice. The gastric mucosa in the vagotomy group was significantly thinner than that of the sham group (Fig. 1f, g); however, body weight showed no difference between the vagotomy and sham groups (Fig. 1h). These results consisted with a previous study^20^, indicating that the unilateral vagotomy was successful.

### Vagotomy suppressed the development of GC in vivo

**Fig. 2:**
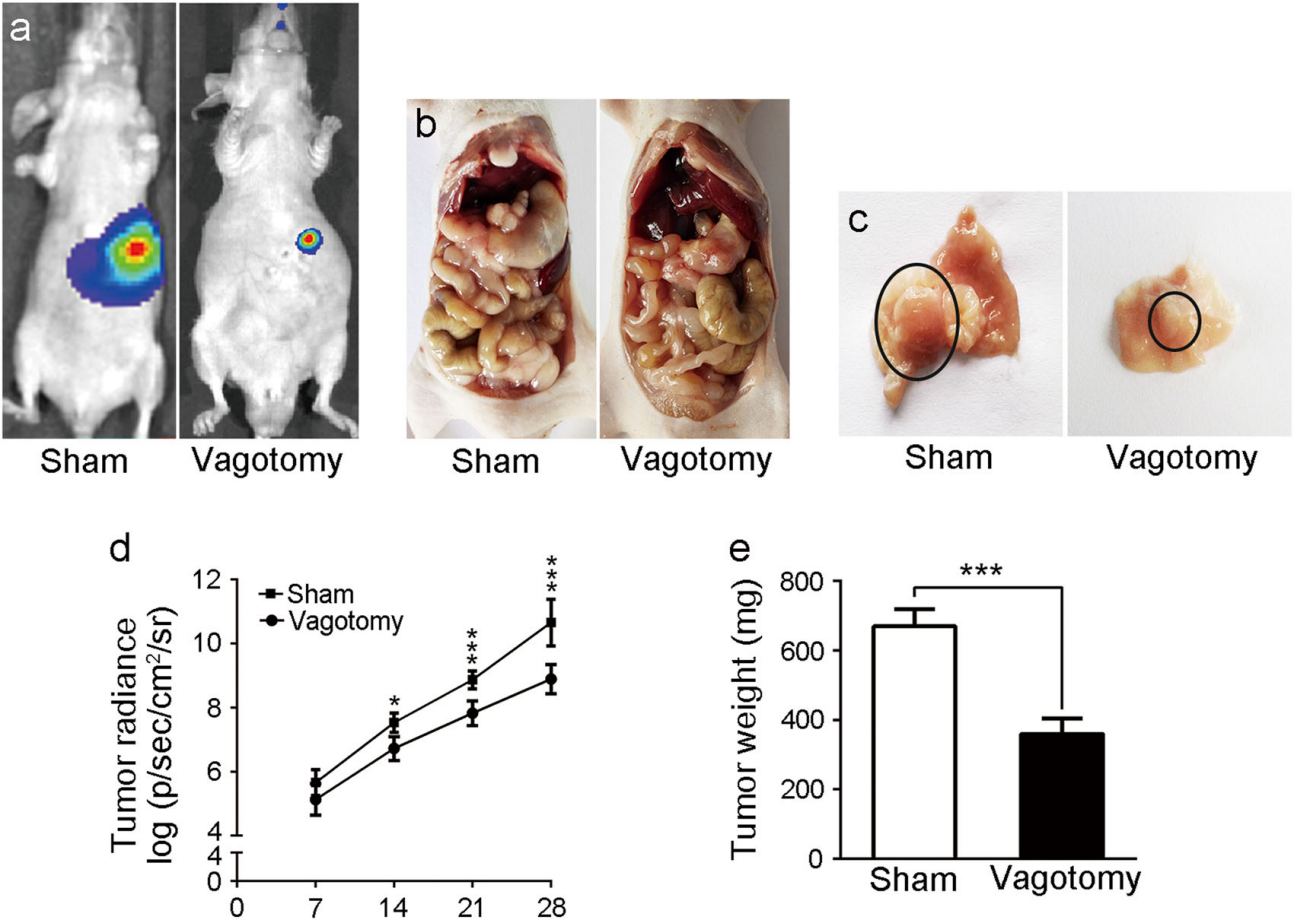
The effects of the vagotomy on the development of GC in the mice. **a** Bioluminescent images of the mice in the sham and vagotomy groups. **b** Anatomic images of the mice in the sham and vagotomy groups. **c** Images of the stomach tumours in the sham and vagotomy groups (black circles show stomach tumours). **d** Tumour radiance on the indicated days in the sham and vagotomy groups. **e** Tumour weight in the sham and vagotomy groups

Two weeks after the nude mice were injected with GC cells in situ, the IVIS imaging system showed that the tumour radiance in the vagotomy group was lower than that of the sham group, and the difference was even more noticeable after three weeks (Fig. 2a, d). To validate this result, we euthanised the nude mice after 4 weeks. We evaluated the harvested tumours, as shown in Fig. 2b, c, e, and found that both the tumour size and weight from the vagotomy group were reduced compared with the sham group. Furthermore, 1 of 6 mice in the sham group exhibited hepatic metastasis, whereas no metastasis was observed in the vagotomy group (Supplementary Fig. 1). Our data indicate that the vagotomy inhibited the development of GC in vivo.

### Vagotomy down-regulated the neural and cancer signalling pathways

**Fig. 3:**
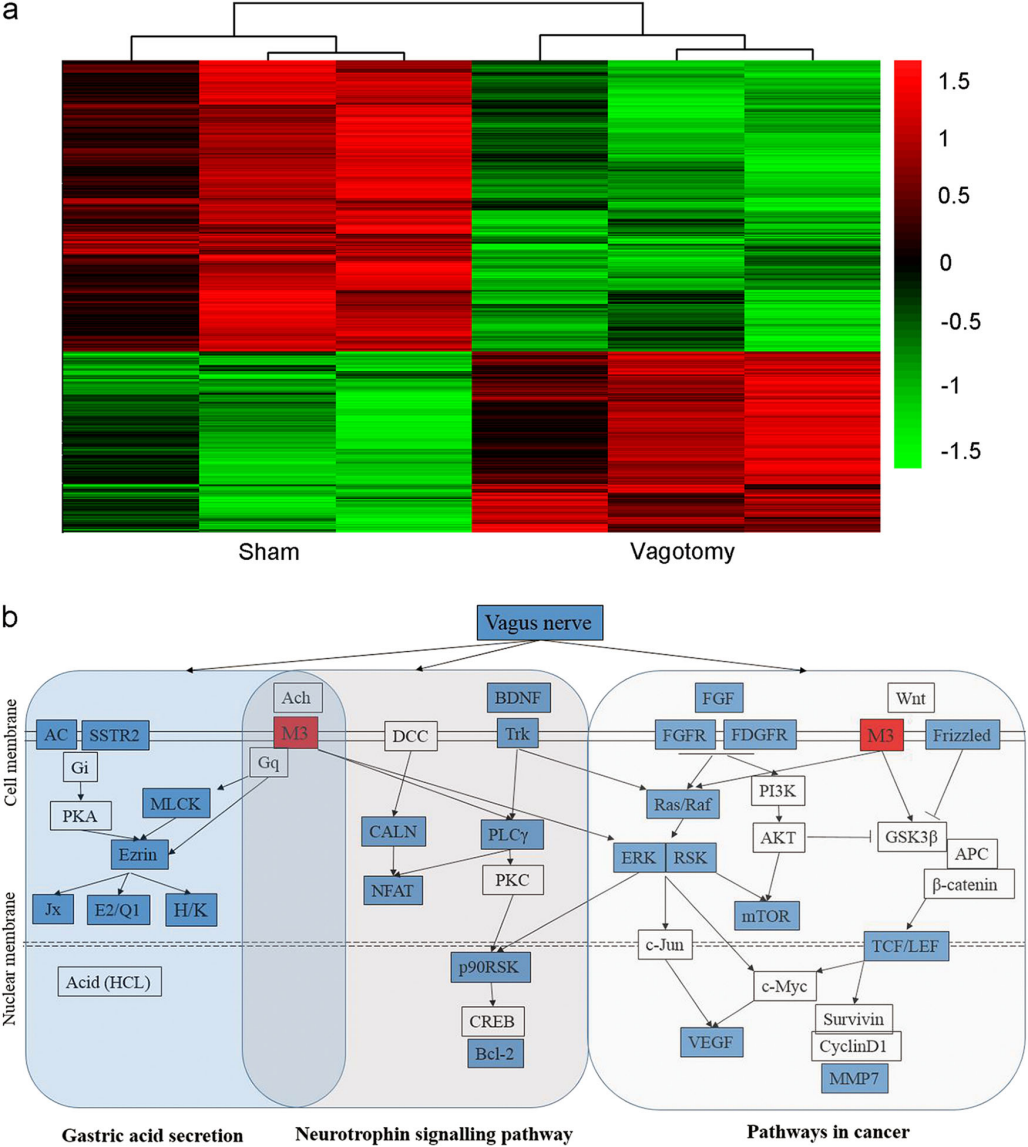
Analysis of significantly altered genes and pathways. **a** Heatmap of significantly altered genes in the sham and vagotomy groups (fold change > 2, *p* < 0.05). **b** Analysis of significantly altered genes and pathways (gastric acid secretion, neurotrophic signalling pathway, pathways in cancer including MAPK signalling, mTOR signalling and Wnt signalling)

**Fig. 4:**
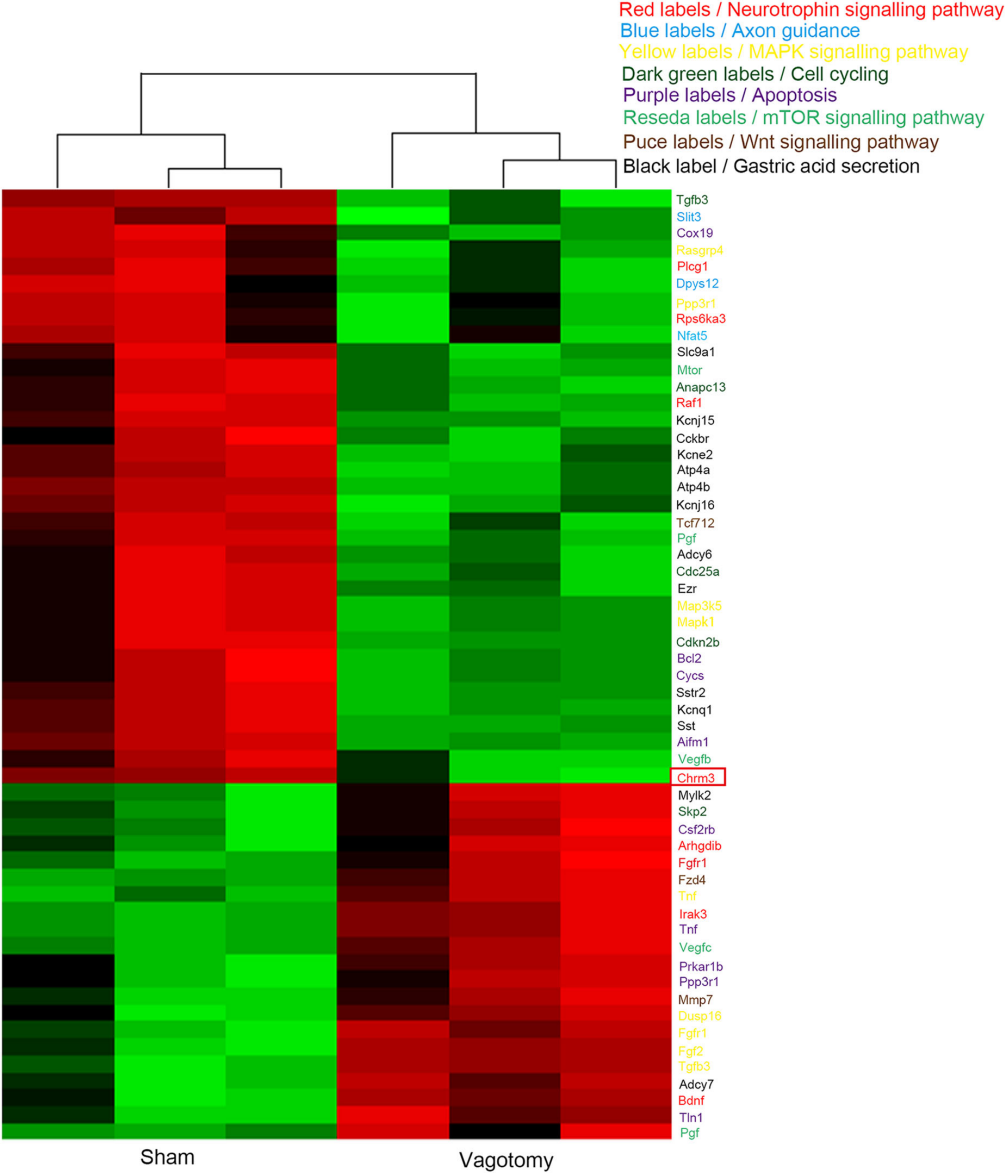
**Heatmap of significantly altered genes involved in the neurotrophic signalling pathway (red labels)**, axon guidance (blue labels), MAPK signalling pathway (yellow labels), cell cycle (dark green labels), apoptosis (purple labels), mTOR signalling pathway (reseda labels), Wnt signalling pathway (dark red labels) and gastric acid secretion (black labels). Red rectangle indicates the Chrm3 gene

To explore the mechanism underlying the vagotomy, we performed gene microarray analysis on both the sham group and the vagotomy group. A total of 1069 genes had higher expression in the vagotomy group than the sham group, while 666 genes showed lower (Fig. 3a). Kyoto Encyclopaedia of Genes and Genomes (KEGG) pathway analysis revealed that some important pathways involved in neural signalling and cancer were altered, including the pathways for neurotrophic signalling, axon guidance, MAPK signalling, cell cycling, apoptosis, mammalian target of rapamycin (mTOR) signalling, Wnt signalling and gastric acid secretion (Figs. 3b and 4). Further analysis identified the key role of M3 in connecting these pathways (Fig. 3b). Cluster analysis of the genes involved in these pathways showed that the neurotrophic signalling, mTOR signalling, Wnt signalling and gastric acid secretion pathways exhibited varying degrees of suppression (Fig. 4). Interestingly, M3 expression level was also decreased in the vagotomy group than the sham group (Fig. 4). These results indicate that M3 might play an important role in how the vagotomy prevents the development of GC.

### M3 mediated the progression of GC affected by the vagotomy

**Fig. 5:**
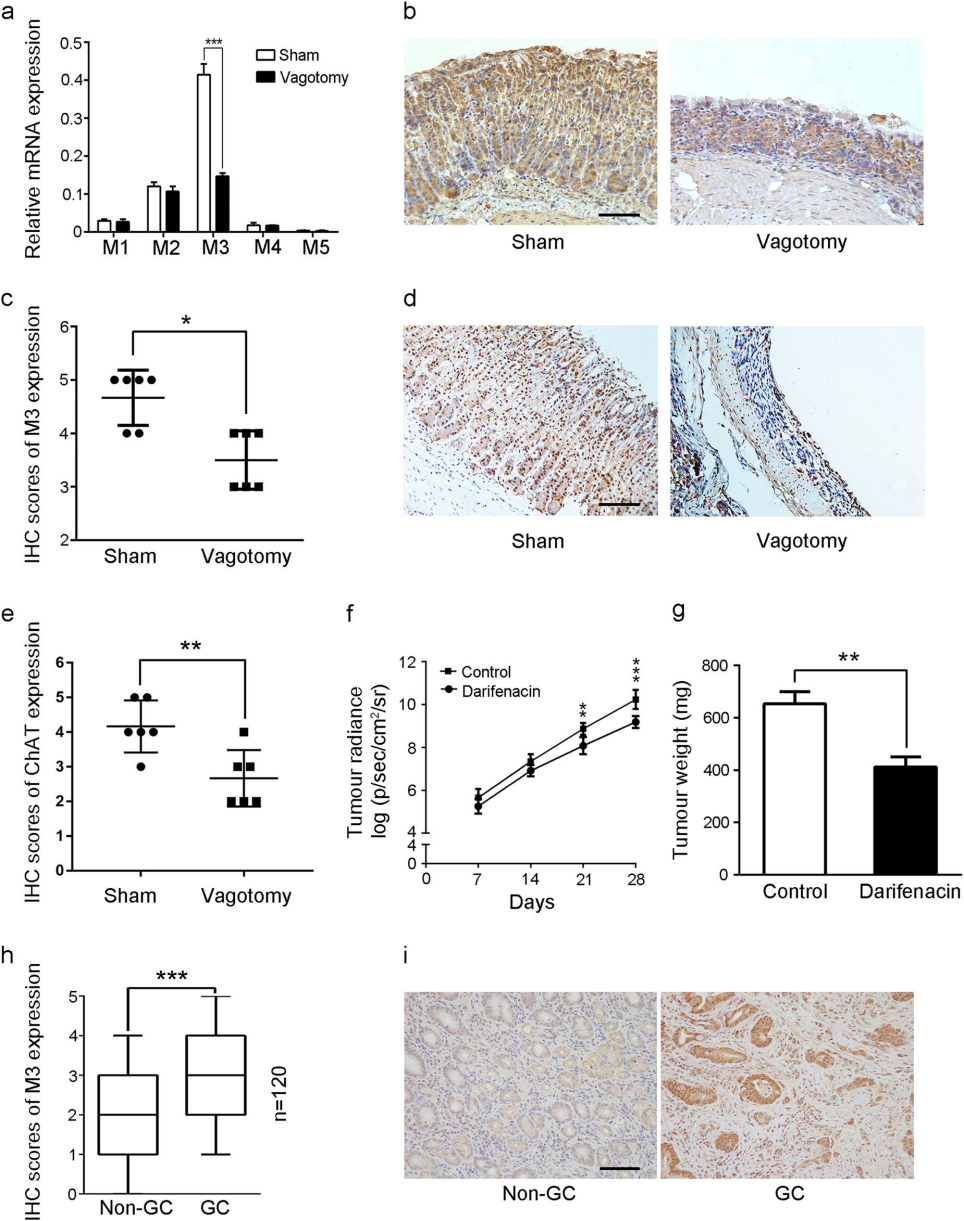
Confirmation of the role of M3 in vagus nerve-induced GC. **a** qRT-PCR analysis of M1, M2, M3, M4 and M5 mRNA in stomach tissues of the sham and vagotomy groups. **b** IHC of M3 in stomach tissues of the sham and vagotomy groups (original magnification, ×100; scale bar = 100 μm). **c** Scatter plot showing IHC scores for M3 protein expression in stomach tissues of the sham and vagotomy groups. **d** IHC of ChAT in stomach tissues of the sham and vagotomy groups (original magnification, ×100; scale bar = 100 μm). **e** Scatter plot showing IHC scores for ChAT protein expression in stomach tissues of the sham and vagotomy groups. **f** Tumour radiance on the indicated days in the control and darifenacin groups. **g** Tumour weights in the control and darifenacin groups. **h** Box plot showing IHC scores for M3 protein expression in 120 human GC tissues and their matched non-cancerous gastric tissues. **i** IHC of M3 in human GC tissues and their matched noncancerous gastric tissues (original magnification, ×200; scale bar = 100 μm)

To identify the role of the muscarinic receptors that regulate the vagus nerve on the development of GC, we performed quantitative real-time polymerase chain reaction (PCR)) to determine the mRNA levels of muscarinic receptors in mouse gastric tissues. The mRNA expression level of M3 decreased in the vagotomy group, while there was no significant difference in M1, M2, M4 and M5 mRNA expression levels between the vagotomy and sham groups (Fig. 5a). We then detected the protein-expression levels of M3 and choline acetyltransferase (ChAT) by immunohistochemistry. The protein-expression levels of M3 and ChAT were decreased in the vagotomy group relative to the sham group (Fig. 5b–e). ChAT is the enzyme necessary for the synthesis of acetylcholine, and M3 is one of the receptors of acetylcholine. These results indicated that vagotomy down-regulated the cholinergic effects in the gastric tissue of mice. To further confirm the functional role of M3, we treated the mice with an M3 selective antagonist (darifenacin, 3 mg/kg/d). Darifenacin suppressed the growth of GC cells in mice (Fig. 5f, g). We then tested the M3 protein levels in 120 paired human GC tissues and non-cancerous gastric tissues by immunohistochemical (IHC) staining. The M3 protein level was significantly higher in the GC tissues than the non-cancerous gastric tissues (Fig. 5h, i), suggesting the functional role of M3 in the development of human GC. Our data confirmed that M3 mediates the progression of the GC, which can be abrogated by a vagotomy.

## Discussion

In this study, we found that the vagus nerve played an important role in the development of GC. Surgical vagotomy inhibited the development of GC in an orthotopic xenograft mouse model. Further analysis showed that multiple signalling pathways participated in this progress, and M3 was a key factor in these pathways. We also demonstrated that an M3 antagonist suppressed the development of GC.

Previous studies showed that denervation suppresses gastric tumourigenesis in insulin–gastrin mice, the N-nitroso-N-methylurea (MNU)-induced GC mouse model and the *Helicobacter pylori* (Hp)-infected H+/K+–ATPase–interleukin-1b GC mouse model^13^. In this study, we used an orthotopic xenograft mouse model with luciferase-labelled MGC-803 cells to mimic the development of GC. MGC-803 cells were derived from a primary poorly differentiated primary mucoid adenocarcinoma of human stomach and the promoting role of cholinergic activity on the proliferation of MGC-803 cells has been proved by our previous study^18^. This model is convenient for monitoring the growth of orthotopic xenograft tumour in stomach. Furthermore, the xenograft tumours in this model exhibited high homogeneity. Therefore, we believe that the orthotopic xenograft mouse model is superior to other models for studying the development of GC.

The vagus nerve has a trophic influence on the stomach. Previous studies showed that a subdiaphragmatic truncal vagotomy inhibited the proliferation of gastric endocrine cells and reduced the weight and height of the gastric mucosa^20,21^. Consistent with previous studies, our data showed that the vagotomy decreased the thickness of the gastric mucosa. Microarray analysis of gene-expression profiles revealed that multiple signalling pathways, including neurotrophic signalling, axon guidance molecules, mitogen-activated protein kinase signalling, gastric acid secretion, apoptosis, cell cycle, mTOR signalling and Wnt signalling, participated in the effects of the vagotomy. These data are in accordance with the result of Zhao’s study^13^. Furthermore, we also established that M3 plays an important role in connecting these pathways.

In a previous study, we demonstrated that M3 contributes to GC cell proliferation and apoptosis^18^. Song et al.^22^ showed that M3 antagonists inhibit small cell lung cancer growth through the extracellular regulated protein kinase/Akt pathway. Cheng et al.^23^ showed that activating M3 receptors promotes the proliferation of colon cancer cells. Raufman et al.^24^ revealed that genetic ablation suppresses the proliferation and neoplasia of murine colon epithelial cell. M3 is an important transmitting element of signal transmission in the vagus nerve^25^. We hypothesised that M3 mediated the effects of the vagus nerve on the development of GC. We first detected the decreases of the M3 mRNA expression and the M3 protein expression in the mice gastric tissues by performing a vagotomy. We also found the decrease of protein levels of ChAT in the mice gastric tissues by performing a vagotomy, indicating that vagotomy down-regulated the expression of ChAT in the gastric tissue of mice. As we know, ChAT is the enzyme necessary for acetylcholine synthesis and M3 is one of the receptors of acetylcholine. The data suggested that vagotomy decreased the secretion of acetylcholine in mice gastric tissues. Our previous study has proved that M3 mediates cell proliferation induced by acetylcholine^18^. Taken together we concluded that vagotomy inhibit the growth of orthotopic xenograft tumour. To further test the functional role of M3 in GC, we treated animals with an M3 antagonist, and the results revealed that the M3 antagonist inhibited the growth of GC in the mice. We also detected M3 protein levels in human paired GC and non-GC tissues, which showed that M3 protein levels were increased in human GC tissues. These data concluded that M3 mediates vagus nerve-induced GC.

In conclusion, we revealed the functional role of M3 in mediating the effects of the vagus nerve on GC. Our study contributes to understanding the mechanism underlying the interaction between GC and the vagus nerve and may help identify new therapeutic targets for GC.

## Materials and methods

### Cell culture, animals and reagents

The human GC cell line MGC-803 was purchased from Cell Centre of Shanghai Biological Sciences Institutes (Shanghai, China). The cells were cultured in RPMI-1640 (Gibco) containing 10% foetal bovine serum (Life Technologies, USA) in a chamber supplemented with 5% CO_2_ at 37 °C. Darifenacin was purchased from Sigma-Aldrich (St. Louis, USA). All animals experiments were conducted under the guidelines of the Nanjing Medical University (NJMU) Institutional Animal Care Committee. Twenty-four4-week-old male BALB/c nu mice were obtained from the Department of Laboratory Animal Centre of NJMU.

### Cell transfection

LV12-U6/Luciferase05/Puro was purchased from GenePharma (Shanghai, China) and packaged into the lentivirus vector. Packaging and transfection of retrovirus were performed per the manufacturer’s instructions.

### Orthotopic xenograft model of GC in nude mice

Four-week-old male BALB/c nu mice were used to establish the animal model of GC. MGC-803 cells transfected with luciferase (suspended in phosphate-buffered saline (PBS)) were injected into the subserosa layer of the lesser curvature of the stomach after intraperitoneal anaesthesia. After 28 days, the IVIS imaging system (Caliper Life Sciences, MA) was used to observe the development of the tumours in situ. After that mice were euthanised, the tumours in situ were harvested and tumour weights were calculated.

### Nude mice vagotomy assay

Twelve 4-week-old male BALB/c nu mice were divided randomly into the sham group and the vagotomy group. For the vagotomy group, the mice were intraperitoneally anaesthetised with chloral hydrate. We made a central abdominal incision exposing the front wall of the oesophagus, and transected the left trunk of the vagal nerve at the subdiaphragmatic level. Finally, the orthotopic xenograft model of GC was constructed. For the sham group, the same procedure was performed, except that the left trunk of vagal nerve was not transected.

### In vivo bioluminescence imaging assay

Mice were anaesthesia by isoflurane and injected D-luciferin (150 mg/kg) intraperitoneal. After 10 min, in vivo bioluminescent image was captured using the IVIS imaging system Bioluminescence photon flux (p/s/cm^2^/steer radiant: photons/s/cm^2^/steer radiant) was analysed in Living Image 4.4 (Perkin Elmer).

### Gene microarray analysis

The tissues were collected from anterior wall of the stomach in the sham group and the vagotomy group at 4 weeks after orthotopic xenotransplantation. The collected mouse stomach samples were kept at −80 °C. Total RNA was extracted by RNAiso according to the manufacturer’s guidelines, and the RNA integrity was determined via the RIN, as assessed with the Agilent Bioanalyzer 2100 (Agilent Technologies, USA). The qualified RNA was further purified by the RNeasy mini kit (Cat. #74106, QIAGEN, Germany) and the RNase-Free DNase Set (Cat. #79254, QIAGEN, Germany). RNA amplification and labelling were performed per the manufacturer’s guidelines. The microarray used was the Agilent Whole Mouse Genome Oligo Microarray (Agilent Technologies, USA). Gene-expression hybridisation slides were scanned by the Agilent Microarray Scanner (Cat. #G2565CA, Agilent Technologies, USA) with the default settings. Data were extracted with Feature Extraction software 10.7 (Agilent Technologies, USA). Raw data were normalised with quantile algorithm using GeneSpring Software 12.6.1 (Agilent Technologies, USA).

### Analysis of significantly altered genes and pathways

Significantly altered genes were identified by their fold change > 2 and *p* < 0.05. Significantly altered pathways were analysed using the KEGG analysis tool from DAVID bioinformatics resources (http://david.abcc.ncifcrf.gov; *p* < 0.1).

### Quantitative real-time PCR

Total RNA was extracted by TRIzol reagent (Invitrogen, USA), and cDNA was synthesised using PrimeScript RT reagent (Takara, Japan) following the manufacturer’s guidelines. The mRNA level of β-actin was used to normalised gene expression. Each PCR test was performed and repeated in triplicate. The primer sequences used are as follows:M1, forward, 5′-TGACCGCTACTTCTCCGTGACT-3′, reverse, 5′-CCAGAGCACAAAGGAAACCA-3′; M2, forward, 5′-TCACAAAACCTCTGACCTACCC-3′, reverse, 5′-TCCACAGTTCTCCACCCTACAA-3′; M3, forward, 5′-CACAATAACAGTACAACCTCGCC-3′, reverse, 5′-GCCAGGATGCCCGTTAAGAAA-3′; M4, forward, 5′-TCGCTATGAGACGGTGGAAA-3′, reverse, 5′-GCTTCTTGACGCTCTGCTTCATTAG-3′; M5, forward, 5′-GAAAGCAGCCCAGACACTGA-3′, reverse, 5′-AGCACAACCAACAGCCCAAG-3′; β-actin, forward, 5′-AGAAAATCTGGCACCACACC-3′, reverse, 5′-TAGCACAGCCTGGATAGCAA-3′.

### IHC analysis

Tissue samples were fixed in 4% formalin, embedded in paraffin and sliced at 4-μm thickness. The slices were incubated with primary antibody of M3 (Santa Cruz Biotechnology; 1:50 dilution) and ChAT (Santa Cruz Biotechnology; 1:100 dilution) overnight and then washed with PBS three times on the second day. After incubating with HRP–polymer-conjugated secondary antibody for one hour at 37 °C, diaminobenzidine was used to develop the colour, and nuclei were lightly counterstained with haematoxylin. Slide views were recorded by microscope (Nikon, Japan) in three random fields. IHC staining scores were assessed based on the staining intensity (0: absent, 1: weak, 2: moderate and 3: strong staining) and the proportion of positive staining cells (0: 10%, 1: 10 to <50% and 2: ≥50% of cells). The IHC scores shows the sum of the proportion and intensity scores.

### In vivo darifenacin assay

Twelve 4-week-old male BALB/c nu mice were divided randomly into the darifenacin group and the control group. For the darifenacin group, darifenacin hydrobromide was dissolved in 50% PBS and 50% DMSO and administered at 3 mg/kg/d subcutaneously for 28 days. For the control group, 50% PBS and 50% DMSO of the same volume was administered subcutaneously for 28 days. The IVIS imaging system was used to observe the tumour development in situ, and the tumour weights were calculated after 28 days.

### Human samples and patients

Human gastric cancer tissues and paired non-cancerous gastric tissues were collected from 120 patients who underwent radical gastrectomy for primary gastric cancer in the First Affiliated Hospital of Nanjing Medical University between 2013 and 2016. The non-GC tissues were collected 5 cm away from the cancer. This study was ratified by the Institutional Ethical Board of the First Affiliated Hospital of Nanjing Medical University. Written informed consents were obtained from patients or their relatives.

### Statistical analysis

Statistical data were analysed in Statistical Product and Service Solutions (SPSS) software version 20.0. The differences were analysed by Student’s *t*-test unless indicated. All data are showed as the mean ± SD and considered significant when *p* < 0.05 (^*^), *p* < 0.01 (^**^), or *p* < 0.001 (^***^).

## Electronic supplementary material

Supplementary figure1

Supp fig 1 legend
