# Supplementary figures and images for: Muscarinic acetylcholine receptor 3 mediates vagus nerve-induced gastric cancer
Source: Oncogenesis. 2018 Nov 21;7(11):88. doi: 10.1038/s41389-018-0099-6 (PMC6246593; doi:10.1038/s41389-018-0099-6)

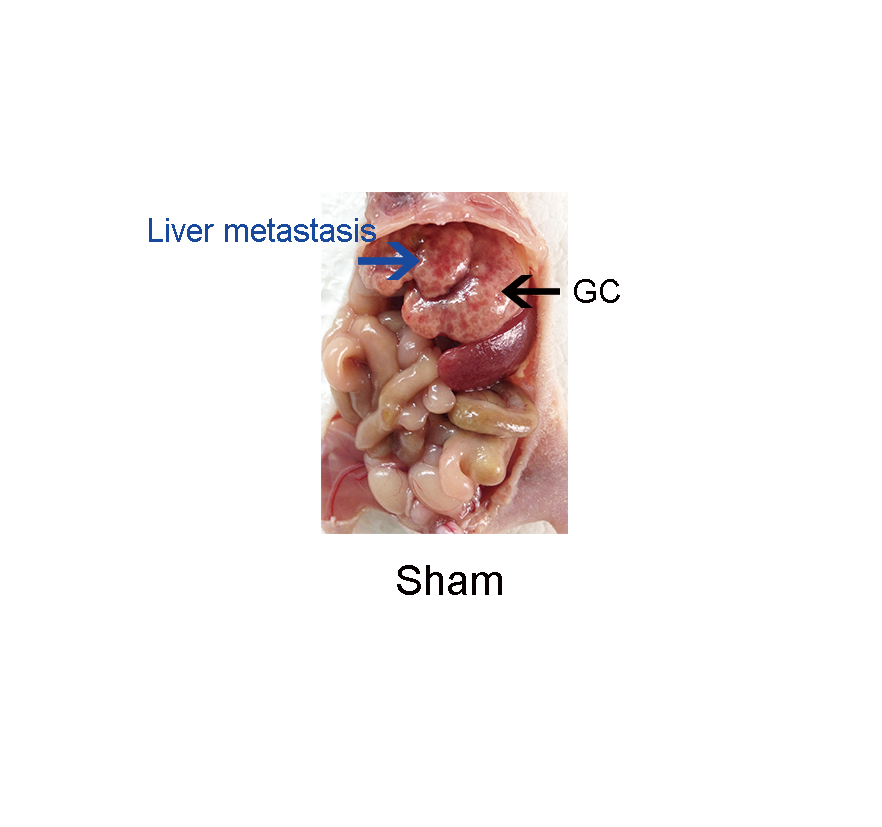

Supplement: Supplementary file 1 — Supplementary figure1 [file 41389_2018_99_MOESM1_ESM.tif]
