# Supplementary material for: Muscarinic acetylcholine receptor 3 mediates vagus nerve-induced gastric cancer
Source: Oncogenesis. 2018 Nov 21;7(11):88. doi: 10.1038/s41389-018-0099-6 (PMC6246593; doi:10.1038/s41389-018-0099-6)
Supplement: Supplementary file 2 — Supp fig 1 legend [file 41389_2018_99_MOESM2_ESM.docx]

**Supplementary figure. 1** Anatomic image of GC and liver metastasis in the sham group (black arrow points to GC; blue arrow points to liver metastasis).
